# The Need to Promote Olfactory Health in Public Health Agendas Across the Globe

**DOI:** 10.1111/coa.70056

**Published:** 2025-11-24

**Authors:** Carl Martin Philpott, Thomas Hummel, Valentina Parma, Matt Lechner, Duncan Boak, Marianna Obrist

**Affiliations:** ^1^ Rhinology and Olfactology Research Group University of East Anglia Norwich UK; ^2^ Department of Otorhinolaryngology, Interdisciplinary Centre for Smell and Taste Technical University Dresden Dresden Germany; ^3^ Monell Chemical Senses Center Philadelphia Pennsylvania USA; ^4^ Division of Surgery and Interventional Science University College London London UK; ^5^ SmellTaste Bicester UK; ^6^ Department of Computer Science University College London London UK

## Abstract

**Background:**

A good sense of smell is essential for physical and mental health, and social wellbeing; however, across the globe, regardless of the setting, national public health agendas never consider smell health. This review aims to summarise the wide‐reaching impact of smell health in public health.

**Methods:**

Narrative review of the literature has been undertaken by leading opinion figures in the field of olfactory health.

**Results:**

The sense of smell should be promoted as an essential pillar of health, as it enables good nutrition and cognitive and psychological well‐being. To improve smell health internationally, a focus on education and awareness, research and targeted public health policies is needed.

**Conclusions:**

We recommend developing smell health educational programmes and awareness campaigns, introducing smell screening and developing and implementing smell health policies across sectors of society. Efforts are needed to ensure equity, diversity and inclusivity for all people, particularly given the current demographic as those seeking help are typically not from a diverse cross‐section of the community.

## Summary

- Olfactory dysfunction is common with negative health consequences.
- There are many medical conditions linked to olfactory dysfunction including neurodegenerative disease.
- Screening programmes are needed to detect olfactory dysfunction given it is a precursor of neurodegenerative disease.
- Education across the medical profession and wider public is needed to promote good smell health and to reduce inequality in those seeking care
- Research to better understand the pathophysiology of olfactory dysfunction and to elicit new therapeutic options is urgently needed.

## 1 Introduction

The World Health Organisation defines health as ‘a state of complete physical, mental and social wellbeing and not merely the absence of disease or infirmity’. A normally functioning sense of smell is an essential pillar of health for the reasons to be explained below. The sense of smell has lagged behind the senses of sight and hearing in terms of its perceived importance and remains the Cinderella sense [1], with its importance still under‐recognised by national public health agendas, educational and research institutions globally. Prior to the emergence of COVID‐19, olfactory (and gustatory) disorders were already a common but under‐rated, under‐researched, and under‐treated sensory loss. Olfactory dysfunction (OD) is present in at least 139 neurological, somatic and hereditary disorders, with evidence suggesting a causal role based on its temporal precedence and prospective predictive power [2]. For instance, increasing evidence has shown that anosmia (complete loss of smell) is an independent risk factor for neurodegenerative disorders [3, 4], increased frailty [5, 6] and reduced longevity (by fourfold) [5, 6, 7] in those acquiring the disorder [6, 8]. The burden of OD and related conditions highlights the current unmet need for accessing healthcare that appropriately recognises the impact of olfactory loss on the quality of life of those affected which is equivalent to other chronic diseases [9, 10].

**FIGURE 1:**
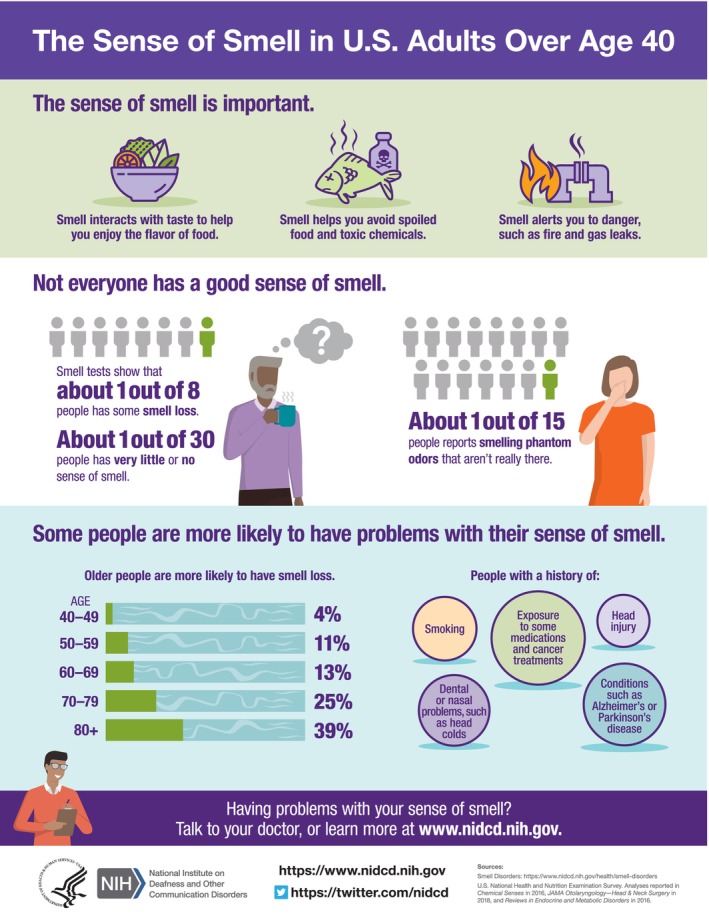
Infographic from the National Institute on Deafness and other Communication Disorders, National Institutes of Health.

The prevalence of OD is underestimated primarily because of the lack of screening or routine testing in secondary care [11], and yet anosmia (loss of sense of smell) is more prevalent than reported rates of profound hearing loss or blindness and affects ~5% of the population. All OD affects about 22% of the population, increasing with age > 60 [12]. Men (especially older men), appear affected to a greater extent than women; yet women with OD seek support more readily with UK/USA data showing poor representation of ethnic minority groups [9, 13]. Aside from ageing, common causes for OD include chronic rhinosinusitis (CRS), infections of the upper respiratory tract including COVID (post‐infectious olfactory dysfunction—PIOD), head trauma (post‐traumatic olfactory dysfunction—PTOD) and other, unexplained cases [14] (see Figure 1 for demographics).

This viewpoint presents a contemporary, evidence‐based, urgent call to action to incorporate smell health into global public health policies. Furthermore, we provide recommendations on strategies and approaches to enhance education, research and the development of policies to improve smell health in all populations, including those who have been historically excluded. We also focus on the links to cognitive decline and reduced longevity for which there is emerging evidence, as called for by the 2023–2027 Strategic Plan of the National Institute on Deafness and other Communication Disorders (NIDCD). The review aims to summarise the wide‐reaching impact of smell health in public health.

## 2 Methods

Global experts of olfaction and olfactory disorders undertook a narrative review to summarise the key aspects of why we believe smell health is a global public health issue. We have categorised the results as global disparities, consequences, linked conditions, inequality and economic burden.

## 3 Results

### 3.1 Conditions Linked to OD

As discussed above, smell health has a far greater impact than it is often credited for, extending to a range of co‐morbidities [2]. Here, we will highlight some of the key topics related to prevention and treatment that merit further consideration.

#### 3.1.1 CRS

Inflammatory sinonasal disease is the leading cause of OD globally and while there are established treatments [15], the olfactory symptoms experienced by patients are the most overlooked, despite being the most concerning to patients themselves [16]. In recent years, monoclonal antibody trials have shown improved health outcomes, especially with respect to olfaction, for those with more severe cases of CRS; yet these drugs continue to remain elusive to otorhinolaryngologists tasked with managing these patients to the great frustration of the patients themselves [16].

#### 3.1.2 Head and Neck Cancer

For patients diagnosed and treated with cancers of the head and neck, typically those of the upper aerodigestive tract, treatment can often involve radical surgery such as a laryngectomy and/or chemo/radiotherapy in the head and neck area. All of these treatment modalities can have a deleterious effect on both smell (and taste) functions in patients during and after treatment [17]. Surgery on the oral cavity and pharynx may also lead to direct removal of gustatory structures and/or injury to cranial nerves. Additionally, both radiotherapy and chemotherapy can subsequently damage olfactory receptors leading to loss of function. For a group of patients in whom nutritional challenges are already present both during treatment and afterwards, the added factor of OD can be further deleterious to health, as described above, and yet remains rather unquantified and under‐addressed.

#### 3.1.3 Neurodegenerative Disease

Despite evidence relating olfactory decline to neurodegenerative disease dating back to 1975, the link between OD and Alzheimer's disease (AD) and Parkinson's disease (PD) remains somewhat neglected, with OD only being considered in the clinical diagnostic criteria for PD in 2015 [18]. Anosmia can predate clinical motor signs of PD by ≥ 5 years with over 90% of patients having OD by the time motor symptoms have manifested [19]. Hypotheses for the neurodegenerative patterns seen in these disorders include α‐synuclein pathology being observed in the olfactory bulb and anterior olfactory nucleus, tau protein propagation from medial temporal lobe structures towards olfactory structures and abnormalities of the primary and secondary olfactory cortices associated with OD in AD; apolipoprotein E4 (APOE‐ℇ4) gene carrier status has a key association for the risk of late‐onset dementia [3]. While a better understanding of the interaction between OD, cognitive decline, APOE‐ℇ4 gene carrier status and neurodegenerative disease is needed, there is clearly an opportunity to leverage OD in population screening.

#### 3.1.4 COVID‐19

The emergence of COVID‐19 as a major respiratory and vascular illness in 2019–2020, also brought with it clear evidence of sudden OD (anosmia/hyposmia) as being a key presenting symptom in patients with COVID‐19 [20]. While the onset of OD during and after viral upper respiratory tract infections (URTIs) such as influenza, parainfluenza, rhinoviruses and endemic coronaviruses is not new [21], the scale of the problem has proved to be much greater. Estimates now show that OD is 8–10‐fold higher in COVID‐19 patients compared to other viral URTIs [22]. With the scale of over 800 million cumulative COVID‐19 cases worldwide, this has created a legacy of an estimated 17 million patients now living with Long‐COVID [23]. While not all of those with Long‐COVID have OD and vice versa, the number of cases of persistent and significant OD is unclear [24], United Kingdom estimates suggest approximately 1 000 000 and higher numbers in the United States [24, 25] may have OD. Within the cluster of symptoms experienced, smell loss and taste loss affect 31% and 24% respectively and those at risk include women, those aged 35–49 years old, of Caucasian ethnicity or those living with disabilities [24, 26]. With memory loss and ‘brain fog’ highly reported in this group and structural brain changes seen in Long‐COVID [24, 27], OD related to Long‐COVID, could be a marker of impending cognitive dysfunction. Further large high‐quality cohort studies are therefore needed to investigate the genetic and biomarker links between cognitive dysfunction, anosmia and severity of acute COVID‐19 infection.

#### 3.1.5 Cardiorespiratory Health

There is now emerging evidence that OD is associated with a 10‐year risk for stroke and long‐term risk for congestive heart failure with a hazard ratio of 1.28–1.32 for poor to moderate OD. What is notable is that this finding was in older adults reporting very good to excellent health [28]. In another study measuring respiratory nasal airflow, anosmic participants demonstrated significantly altered patterns in wake and in sleep. Given the link between respiratory patterns and health, emotion and cognition, the authors suggest that the negative impacts of OD may also be indirectly related through altered breathing [29].

### 3.2 Consequences of OD

#### 3.2.1 Domestic and Work Hazards

Domestic hazards such as the inability to detect gas/fire/smoke, eating spoiled food and exposure to hazards in the workplace are common risks in those with OD; Lee et al. showed that 86% worried about safety and that over a 5‐year period, common hazards encountered included ≥ 1 spoiled food incident (food poisoning) in 32%, ≥ 1 gas incident in 15%, ≥ 1 gas scare in 35% and ≥ 1 work incident in 19% [30]. While the installation of smoke and carbon monoxide detectors is mandatory in both rented properties in the United Kingdom and in the United States, there is no legal requirement to install natural gas detectors or for those affected to carry any personal worn detectors.

#### 3.2.2 General Well‐Being and Psychiatric Health

Individuals with OD face a significant quality‐of‐life burden [31], including high rates of eating disorders (92%), social isolation (57%), relationship difficulties (54%), anxiety (45%) and depression (43%) [32]. This psychological toll is often trivialized, despite depression rates exceeding the global average of 4% [33]. Measured by EuroQol 5‐Dimension 5‐Level (EQ‐5D‐5L), its associated Visual Analog Scale (EQ VAS), and the Beck Depression Inventory, their perceived health status aligns with chronic conditions such as type 2 diabetes, asthma, and age‐related eye disease [10].

#### 3.2.3 Nutritional Health

Accumulating evidence shows the impact of OD on diet quality and nutrition status, worsened if smell distortion (parosmia) coexists with quantitative smell loss (anosmia or hyposmia, partial smell loss) [34]. SATD patients eat a more energy‐dense, high fat/saturated and fat/added sugar and less varied diet [34, 35] with obesity and micronutrient deficiency as potential consequences [36]. Again, given that global figures show 43% of adults are overweight and 16% are obese [37], smell health may have a significant impact on this dimension of the global health burden. In a specific group of patients with type 2 diabetes mellitus, the prevalence of OD was 71% in those with diabetic complications [36]. Patients with type 1 diabetes mellitus show similar complications, with long‐ranging effects into adulthood [38].

### 3.3 Inequality and Economic Burden in Smell Health

Recent work around equality, diversity and inclusion with respect to OD has identified several key factors about limitations of access to care for non‐Caucasians in the United Kingdom and elsewhere. Discrimination, cultural differences in the perceived importance of smell and taste, language barriers, unfamiliarity with healthcare systems and financial constraints were all cited by those in ethnic minority communities [39]. Currently there is a paucity of high‐quality evidence supporting treatment options [14] leaving OD patients with limited therapy or empathy [9, 40] and with the above prevalence estimates, there is a vast unmet need for healthcare systems and society. Data on the management of the psychological impact suggests that OD contributes to an estimated annual drug cost of 8.3 million antidepressant prescriptions in the United Kingdom and billions in the United States. Additionally with the scale of dementia (e.g., 1 000 000) people in the United Kingdom costing £25 billion per year [41], this highlights the potential for the link to olfactory decline to be exploited in screening for and managing OD‐associated dementia and reduced longevity in the emerging burden of our ageing populations.

### 3.4 Global Disparities in Smell Health Awareness

Beyond COVID, for which there is a substantial burden of OD (see above), there is a paucity of awareness specifically on the impairment of smell in relation to poor health and health risk behaviours, so much so that the perception is now that ‘COVID = smell loss’ and ‘no COVID = no smell loss’. This sits in contrast with the WHO Global Burden of Disease website, which states that ‘policy interventions to date have been insufficient to address rising exposure to risk factors [for poor health] including high body mass index, high blood sugar, ambient air pollution, drug use, and high temperatures’ [42]. Smell dysfunction is associated with all these risk factors and as a potential barometer for environmental pollution, OD may well be closely linked to these risk factors. Nonetheless, the US Centers for Disease Control and Prevention, for example, fails to list anything relating to smell and taste disorders (SATDs) or their symptoms and a European Union Directive cites creating a toxin‐free environment as a priority [43]. Given the wide‐reaching health impacts, the lack of global public health agendas appears to be an enormous omission.

## 4 Discussion: Recommendations to Promote Smell Health Across the Globe

### 4.1 Education and Awareness

The medical profession typically receives little or no education on the topic of smell and taste and their related disorders, although in the UK anosmia has recently been listed as one of the symptoms that medical students passing the medical licensing assessment are expected to be familiar with, including doctors qualifying outside of the United Kingdom that come to work in the United Kingdom [44]. However, globally there appears to be no curriculum consensus and the likelihood of inclusion of the topic in medical undergraduate programmes is low to non‐existent. This is further exacerbated by the lack of expertise to deliver this education given the paucity of experts in the field internationally, especially outside of western countries. An exception is the National League for Nursing in the United States, which is currently developing a curriculum to integrate chemosensory science into undergraduate and graduate curricula. Work by the charity SmellTaste (formerly Fifth Sense) has frequently demonstrated a lack of empathy received by those with OD when approaching medical professionals for advice and support, often being given a shrug or told that they should feel relieved that at least they are not blind or deaf [9, 40]. Sadly, this experience is heard internationally and even within the specialty of Otorhinolaryngology which typically receives the secondary and tertiary referrals.

Education of all medical students, healthcare professionals and social workers on the topic of OD and related disorders, as well as a basic understanding of the related anatomy and physiology, should be a prerequisite in all curricula and a cross‐specialty topic for continuous medical education. While specialists in Otorhinolaryngology and Neurology can be targeted with educational programmes, educating medical students will be the easiest and quickest way to ensure that all medical practitioners are at least familiar with the topic.

### 4.2 Research

Despite recent efforts to prioritise research needs for OD [45] there remains a paucity of basic science and high‐quality clinical research to develop knowledge and treatments. While the charities for OD such as Fifth Sense and the Smell and Taste Association of North America (STANA) have been leading efforts to elevate the research agenda, there is a need for greater investment in both the basic sciences and the clinical domains relating to OD. There is also an opportunity to encourage interdisciplinary research through collaboration with computer science, advances in digital health technology and increasingly the integration of AI for personalised preventive health and care [46].

### 4.3 Public Health Policies

OD prospectively predicts cognitive and cardiovascular health as well as all‐cause mortality and therefore global healthcare systems should look to implement public health policies through screening, testing and smell care. Access to olfactory testing should become akin to that for sight and hearing testing. Smell care denotes the opportunity to promote olfactory training to help reduce cognitive decline but has to be linked to smell testing as many people will be unaware of a gradual decline in olfactory function without prompting. It is also vital that efforts to develop effective interventions for OD are accelerated given the current paucity available.

## 5 Conclusion

We have demonstrated that OD affects a large proportion of the global population, in significant ways and that OD presents an unutilised opportunity for screening for general health. We hope that through this publication, the international public health agenda may begin to consider the sense of smell as an important item for inclusion in public health policy.

## Author Contributions

The viewpoint was conceived by M.O. and C.M.P. All authors have contributed to the content of the manuscript.

## Funding

The project ‘I‐smell: Engaging Users in Smell Self‐Care at Home’ on the Feasibility of Engaging Users With Daily Digital Smell Training at Home was funded by the Engineering and Physical Sciences Research Council, UK.

## Ethics Statement

No ethical approval was required for this review.

## Conflicts of Interest

D.C.B. is the CEO of the charity Fifth Sense and C.M.P. is the chair of the board of trustees for the charity. The other authors declare no conflicts of interest.

## Data Availability

Data sharing not applicable to this article as no datasets were generated or analysed during the current study.
